# Bioactive scaffolds in stem-cell-based therapies for cardiac repair: protocol for a meta-analysis of randomized controlled preclinical trials in animal myocardial infarction models

**DOI:** 10.1186/s13643-018-0845-z

**Published:** 2018-12-05

**Authors:** Kashif Khan, Karina Gasbarrino, Ibtisam Mahmoud, Georges Makhoul, Bin Yu, Line Dufresne, Stella S. Daskalopoulou, Adel Schwertani, Renzo Cecere

**Affiliations:** 1 Division of Cardiology and Cardiac Surgery, McGill University Health Centre, Montreal, Quebec Canada; 2 Division of Experimental Medicine, Department of Medicine, Faculty of Medicine, McGill University Health Centre, Montreal, Quebec Canada; 3 McConnell Resource Centre, McGill University Health Centre, Montreal, Quebec Canada; 4 Glen Campus-The Royal Victoria Hospital, 1001 Decarie Blvd, Block C, C07.1284, Montreal, Quebec H4A 3J1 Canada

**Keywords:** Stem cells, Scaffold, Hydrogel, Patch, Myocardial infarction, Animal, Meta-analysis, Systematic review

## Abstract

**Background:**

Acute myocardial infarction (MI) remains one of the leading causes of death worldwide with no curative therapy available. Stem cell therapies have been gaining interest as a means to repair the cardiac tissue after MI and prevent the onset of heart failure. Many in vivo reports suggest that the use of stem cells is promising, yet clinical trials suggest that the cells fail to integrate into the native tissue, resulting in limited improvements in cardiac function and repair. To battle this limitation, the combination of using stem cells embedded in a bioactive scaffold that promotes cell retention is growing in interest. Yet, a systematic review of the literature on the use of stem cells embedded in bioactive scaffolds for cardiac repair has not yet been performed. In this protocol, we outline a systematic review and meta-analysis of preclinical trials in animal MI models that utilize stem cell-embedded scaffolds for cardiac repair and compare their effects to stem cell-treated animals without the use of a scaffold.

**Methods/design:**

We will search the following electronic databases: Cochrane Library, MEDLINE, Embase, PubMed, Scopus and Web of Science, and gray literature: Canadian Agency for Drugs and Technologies in Health and Google Scholar. We will only include randomly controlled preclinical trials that have directly investigated the effects of stem cells embedded in a scaffold for cardiac repair in an animal MI model. Two investigators will independently review each article included in the final analysis. The primary endpoint that will be investigated is left ventricular ejection fraction. Secondary endpoints will include infarct size, end systolic volume, end diastolic volume, fractional shortening and left ventricular wall thickness. Pooled analyses will be conducted using the DerSimonian-Laird random effects and Mantel-Haenszel fixed-effect models. Between-studies heterogeneity will be quantified and determined using the Tau^2^ and *I*^2^ statistics. Publication bias will be assessed using visual inspection of funnel plots and complemented by Begg’s and Egger’s statistical tests. Possible sources of heterogeneity will be assessed using subgroup-meta analysis and meta-regression.

**Discussion:**

To date, the use of scaffolds in myocardial repair has not yet been systematically reviewed. The results of this meta-analysis will aid in determining the efficacy of stem cell-embedded scaffolds for cardiac repair and help bring this therapy to the clinic.

**Electronic supplementary material:**

The online version of this article (10.1186/s13643-018-0845-z) contains supplementary material, which is available to authorized users.

## Background

The burden of heart failure afflicts approximately 6.5 million Americans over the age of 20 [1]. It is characterized as the inability of the heart to meet the metabolic demands of the body [2]. Although there are several types of heart failure, the most common is left ventricular heart failure, generally caused by myocardial infarction (MI). The immediate result of MI is the death of cardiomyocytes, fibroblasts, and endothelial cells [3]. The heart attempts to compensate by increasing the workload on the neighboring cells. This compensatory mechanism is helpful at first; however, overtime it leads to maladaptive cardiac remodeling and progression towards heart failure [4–6]. With no curative therapies available, heart failure patients are forced to use left ventricular assist devices or undergo heart transplantation, both of which require very invasive surgeries with significant morbidity and mortality [7].

In an attempt to find innovative therapies for heart failure patients, researchers have been investigating the use of stem cells for cardiac repair over the past 18 years [8]. It is well understood that direct injection of stem cells result in acute improvements in left ventricular ejection fraction (LVEF), scar size, and left ventricular remodeling [8]. Yet, the results from clinical trials indicate only modest improvements in cardiac function [8, 9]. One of the major reasons for these limited outcomes is insufficient cell engraftment in the cardiac tissue [10, 11]. Several strategies are currently being explored to battle this limitation; however, one of the most promising therapies is to embed cells in a bioactive scaffold, a 3D structure that promotes cell retention, growth, and tissue repair [12–14], and implant this cell-scaffold structure into infarcted hearts.

There are two types of scaffolds that have been used in cell-based therapies for cardiac repair: hydrogels and patches [14]. Several naturally occurring compounds are utilized to generate hydrogels such as chitosan, collagen, decellularized tissues, fibrin, hyaluronic acid, keratin, Matrigel, and synthetic polymers composed of either polyethylene glycol or poly(N-isoproylacrylaminde are also being investigated [15]. One of the major advantages of using hydrogels for cardiac repair is that they are injectable and can be administered using minimally invasive procedures, such as epicardial, intracoronary, and endocardial injections [15]. Preclinical trials in animal MI models using stem cell-embedded hydrogels have shown an attenuation of ventricular remodeling and inflammation while improving ejection fraction and reducing scar size [16–18]. Interestingly, a recent clinical trial found that severe heart failure patients injected with an alginate-based hydrogel, coupled with standard therapy, improved peak VO2 and time in a 6-min walk test after 6 months of therapy [19], indicating that hydrogel-based therapies alone can be used to treat heart failure patients. Several materials for cardiac patches have also been identified; these include collagen, fibrin, decellularized tissues, and synthetic polymers such as polygycolic acid, poly ε-caprolactone-co-l-lactide, poly-glycerolsebacate, and polydimethylsiloxane [20]. Although the use of these patches require open heart surgery, it is likely that minimally invasive approaches will be explored in the future [21]. Preclinical trials suggest that patches promote cardiogenic differentiation, cell retention, and proliferation while reducing scar formation [21–23]. Moreover, a recent clinical trial utilized a fibrin-based patch embedded with differentiated human embryonic stem cells and reported a 36% improvement in LVEF [24, 25].

The use of bioactive scaffolds for cardiac repair is a promising therapy, yet, a systematic review of the literature has not yet been conducted. In this protocol, we outline a meta-analysis to summarize the current evidence on the use of stem cells in bioactive scaffolds for cardiac repair in animal MI models. The results of this study will aid in understanding the benefits of using scaffolds for cardiac repair and bringing this therapy to the clinic.

## Methods/design

### Protocol

This protocol was written in accordance with the preferred reporting items for systematic reviews and meta-analysis protocols (PRISMA-P) guidelines (Additional file 1) [26] and is adapted from the structure provided in the Systematic Review Protocol for Animal Intervention Studies [27]. This protocol will be registered in PROSPERO after feedback and peer review.

### Specify the disease of interest

Due to the inherent limitations of adult cardiomyocytes, the cardiac tissue becomes irreversibly necrosed after an MI [28], with approximately 50% of heart failure patients dying within 5 years from their MI [29]. The pre-clinical model for MI in humans is coronary artery ligation of the left descending coronary artery in animals, leading to either partial or complete obstruction of blood flow to the left ventricle.

### Specify the population studies

Bioactive scaffolds have been used in several animal MI models. Therefore, we will include all preclinical studies that use the following animals: rats, mice, pigs, rabbits, goats, dogs, and sheep.

### Specify the intervention

The intervention will consist of stem cell-based therapies for cardiac repair with or without a bioactive scaffold. Stem cells are defined as undifferentiated cells that are clonogenic and can differentiate into other cells types. Studies will be included if they utilize one of the following scaffolds: hydrogels or patches.

### Specify the control population

Studies will be included only if they provide data for a suitable cell-based control. Sham animals or saline/PBS-injected animals will be excluded from the analysis.

### Specify the outcome measures

Primary endpoint: LVEF.

Secondary endpoints: infarct size, end systolic volume, end diastolic volume, fractional shortening, and left ventricular wall thickness.

### Research question

Do scaffolds provide better functional outcomes for cardiac repair than a stem cell-only approach?

### Identify the literature databases and search terminology

We will search the following electronic databases: Cochrane Library, MEDLINE, Embase, PubMed, Scopus and Web of Science, and gray literature: Canadian Agency for Drugs and Technologies in Health and Google Scholar. The search terminologies that will be utilized are described in Table 1.

**Table 1:** Search terminology to be used in the meta-analysis

#	Term
1	Tissue Scaffolds/
2	exp Cell Engineering/
3	(scaffold* or hydrogel* or patch* or bead* or biomaterial*).tw,kf.
4	(tissu* adj3 engineer*).tw,kf.
5	1 or 2 or 3 or 4
6	exp Stem Cells/
7	Myocytes, Cardiac/
8	Myocardium/cy [Cytology]
9	limit 8 to yr. = “1968–2002”
10	Cell Differentiation/
11	limit 10 to yr. = “1966–1983”
12	Cell Line/
13	limit 12 to yr. = “1969–1983”
14	Cells, Cultured/
15	limit 14 to yr. = “1972–1983”
16	Colony-Forming Units Assay/
17	limit 16 to yr. = “1979–1983”
18	((progenitor* or cardi* or heart* or stem* or mother) adj5 (cell* or myocyt*)).tw,kf.
19	(cardiomyocyt* or cardio myocyt* or cardiac myocyt*).tw,kf.
20	or/6–19
21	5 and 20
22	exp Myocardial Infarction/
23	(MI or AMI or STEMI or NSTEMI or ((myocard* or myo-card* or heart* or cardiac* or subendocard* or coronary) adj3 (infarct* or reinfarct* or attack*))).tw,kf.
24	(MACE or ((major or acute) adj1 ((cardiac* or cardio* or coronary) adj1 event*))).tw,kf.
25	22 or 23 or 24
26	21 and 25
27	Regeneration/
28	(repair* or regen* or heal* or preserv*).tw,kf.
29	27 or 28
30	26 and 29
31	Animals/ not (Animals/ and Humans/)
32	((animals or animal or canine* or cat or cats or dog or dogs or feline or goat* or hamster* or mice or monkey or monkeys or mouse or murine or pig or pigs or piglet* or porcine or primate* or rabbit* or rats or rat or rodent* or sheep*) not (human* or patient*)).ti.
33	31 or 32
34	30 and 33
35	remove duplicates from 34

### Other sources

We will also analyze the reference lists of previous meta-analyses and reviews to further identify relevant studies. Reference lists of eligible studies will also be reviewed.

### Study selection protocol

Two independent reviewers (K.K. and K.G) will screen through each article via two phases:

1. Title/abstract screening
2. Full-text screening

Both reviewers will attempt to agree on the studies included in the meta-analysis. If no agreement can be reached, a third reviewer (R.C.) will be consulted.

### Reporting of results

Results of the meta-analysis will be reported in accordance with the PRISMA guidelines [26].

### Study selection criteria

#### Type of study design

Inclusion criteria: randomized, controlled trials.

Exclusion criteria: non-randomized, non-controlled trials, reviews, case studies, and editorials.

#### Type of disease model

Inclusion criteria: animal MI models through temporary or permanent occlusion of the coronary arteries. There will be no restrictions in terms of time and type of coronary occlusion.

Exclusion criteria: co-intervention studies.

#### Type of intervention

Inclusion criteria: studies utilizing a scaffold for stem cell-based cardiac repair. There will be no restrictions on the type of scaffold or stem cell used.

Exclusion criteria: non-scaffold-based trials, non-stem cell trials.

#### Type of outcome measures

Inclusion criteria: The primary endpoint for this study will be LVEF by echocardiography. If the study mentioned the use of echo but did not report the data for any of the primary or secondary outcomes measures, the authors will be contacted via email to provide this information. If authors do not respond within 4 weeks of initial contact, a second email will be sent. In parallel, the authors will also be contacted via several research platforms: ResearchGate, Academia, Loop and Quora. If authors do not respond after 4 weeks of the second contact, the study will be excluded from the analysis. Only studies reporting means and standard deviation or standard error of the mean will be included.

Exclusion criteria: studies that did not measure LVEF by echocardiography or if missing data was not provided by the authors after email contact.

### Language restrictions

No language restrictions will be placed when searching for studies.

### Publication date restrictions

No date restrictions will be placed when searching for studies.

### Order for a priori title/abstract screening

1. No original data
2. No stem cell treatment
3. No scaffold
4. No animal models
5. No MI

### Order for a priori full-text screening

1. No original data
2. No stem cell treatment
3. No scaffold
4. No animal models
5. No MI
6. LVEF not reported
7. No randomization
8. No suitable control

### Study characteristics to be extracted

Study characteristics will be extracted from selected articles using a standardized proforma criteria (Table 2).

**Table 2:** Study characteristics to be extracted using standardized proforma

Characteristic	Details to be extracted
Study citation	DOI, complete author list, publication year, journal, funding source, geographical location of study
Study design	Randomization method, number of animals per group
Animal model	Animal species, strain, sex, weight, age
MI model	Permanent ligation or ischemia-reperfusion injury, duration of coronary occlusion
Intervention	Type of scaffold, type of stem cell, cell characteristics, dosage, delivery technique, duration of follow-up
Result	LVEF, infarct size, end systolic volume, end diastolic volume, fractional shortening, left ventricular wall thickness

### Outcomes and units of measurement

1. LVEF as %
2. End systolic volume in mL
3. End diastolic volume in mL
4. Infarct size as %
5. Left ventricular wall thickness in mm
6. Fractional shortening as %

### Risk of bias assessment

Risk of bias will be measured using the SYRCLE’s risk of bias tool (Table 3) [30].

**Table 3:** SYRCLE’s risk of bias tool

Type of bias	Domain
Selection bias	Sequence generation Baseline characteristics Allocation concealment
Performance bias	Random housing Blinding
Detection bias	Random outcome assessment Blinding
Attrition bias	Incomplete outcome data
Other sources of bias	

### Methods of data extraction and retrieval

Relevant data will be extracted using a standardized proforma. Data will be preferentially extracted from result tables in the selected manuscripts as mean ± standard deviation. If only individual data is present in the manuscript, the mean and standard deviation will be calculated from these values. If the data is not listed in the table, the text in the results section will be carefully read for any important information. If the data is only available from graphs, the data will be extracted manually using the Image J® software version 1.47t (ImageJ, US National Institutes of Health, Bethesda, MD, http://imagej.nih.gov/ij/, 1997–2015). If the data is not available in the text, but mentioned throughout the manuscript, the authors will be contacted via email.

### Data synthesis/analysis

#### Data combination

A systematic review of the literature will be performed, combined into forest plots and meta-analyzed.

#### Specify the criteria for combining data

The overall analysis will combine the data from the use of all bioactive scaffolds and compared with cell-only controls. Data will be further analyzed by subgroup analysis based on three major criteria: type of scaffold, stem cell, and animal model. Therefore, a separate subgroup meta regression will be performed for these criteria.

#### Specify the outcome measurements

1. LVEF: raw mean difference
2. End systolic volume: standardized mean difference
3. End diastolic volume: standardized mean difference
4. Infarct size: standardized mean difference
5. Left ventricular wall thickness: standardized mean difference
6. Fractional shortening: raw mean difference

#### Specify possible sources of heterogeneity

1. Scaffold type (hydrogels, patches)
2. Cell type (embryonic, mesenchymal, cardiac progenitor)
3. Cell characterization through gene/protein expression analysis
4. Animal characteristics (species, sex, age, weight, comorbidities)
5. Type of MI model (permanent ligation, ischemia-reperfusion injury)
6. Duration of MI model
7. Follow-up
8. Randomization
9. Blinding
10. Immunosuppressive therapy

#### Specify the statistical model

Pooled analyses will be conducted using the DerSimonian-Laird random effects or Mantel-Haenszel fixed-effect models. In case of statistically significant between-studies heterogeneity, random-effect models will be applied [31–33]. Data will be expressed as mean differences (MD) with 95% CI and considered significant at *P* < 0.05. Forest plots will be used to display the relative treatment effect and its 95% CI for each trial, scaffold type, cell type, animal model, and for the overall random-effects meta-analyses. Data will be analyzed using Review Manager (RevMan) 5.3 (The Nordic Cochrane Centre, The Cochrane Collaboration, Copenhagen, Denmark) for primary analyses. Meta-regressions will be performed to assess the significance of subgroup effects with STATA software, v13 (StataCorp, College Station, TX) with a significance level set at *P* < 0.05. Publication bias will be assessed by visual inspection of funnel plots and formally complemented by Begg’s and Egger’s statistical tests [34], where *P* < 0.05 was considered evidence of small study effects. Between-studies heterogeneity will be quantified and determined via the Tau^2^ and *I*^2^ statistics with the significance level set at *P* < 0.10 [33]. Sources of heterogeneity will be explored by sensitivity analysis via systematic removal of individual trials.

### Possible foreseen limitations

It may be likely that the studies that fit the inclusion criteria may be too heterogeneous, as determined quantitatively by the Tau^2^ and *I*^2^ statistics. In the event that this occurs, DerSimonian-Laird random effects analysis will be performed.

## Discussion

Meta-analyses are a necessary step in bringing novel therapies to the clinic, as they help to quantitatively review the literature, while systematically exploring validity and bias [33]. In innovative field, meta-analyses have been widely used to understand controversial findings in clinical and preclinical trials that use stem cells for cardiac repair [8, 27, 35–37]. To add to this growing field of research, we provide a protocol of a systematic review and meta-analysis that explores the use of bioactive scaffolds in stem cell-based therapies for cardiac repair. To our knowledge, this is the first meta-analysis that directly investigates the combination of stem cells and bioactive scaffolds for cardiac repair.

The use of bioactive scaffolds is a promising therapy to improve the retention of exogenous stem cells in the cardiac tissue. However, other therapies also exist. Since only a small number of cells are thought to remain in the diseased cardiac tissue after a single dose injection, repeated cell administration strategies are also being utilized to enhance this effect [38]. Rats with induced MI given 3 doses of stem cells 14 days apart demonstrated larger improvements in left ventricular function compared to a single administration [39]. Similar to drug therapies, it is thought that the repeated administration of stem cells has a cumulative effect on cardiac function [38]. Another strategy to improve stem cell retention is to use cells that are differentiated towards the cardiac lineage, also known as cardiac progenitor cells (CPCs) [40]. CPCs are able to self-renew and differentiate into endothelial cells, smooth muscle cells, and fibroblasts, and are characterized by several cardiac markers such as c-kit, SSEA-1, Sca-1, and Isl-1 [41]. In fact, several CPC populations reside in the native adult heart; however, these cells are not well characterized and remain difficult to isolate. A recent meta-analysis investigated the use of cardiac progenitor cells for cardiac repair after MI in preclinical trials and reported significant improvements in LVEF compared to non-cardiac progenitor cell controls [35]. Cardiosphere-derived cells (CDCs), cardiac explants that have aggregated to form a sphere of proliferating CPCs [42], were found to promote the greatest improvements in LVEF [35]. In addition, a clinical trial using autologous CDCs in patients after MI resulted in significant reductions in scar mass and improved regional contractility, and regional systolic wall thickening [43]. Given the recent boom in research on these novel therapies for cardiac repair, it is likely that more clinical trials will arise in the future.

In fact, a recent clinical trial combined the approach of using embryonic stem cell-derived CPCs embedded in a patch for cardiac repair [24]. Embryonic stem cells were differentiated using a two-factor approach, and the differentiated population was characterized appropriately [25]. These differentiated CPCs were then embedded in a fibrin-based patch and delivered to patients undergoing coronary artery bypass surgeries after MI. The study reported that all patients had improved symptoms with significant improvements in wall motion in the treated area [25]. Thus, it is likely that a combination approach that utilizes both CPCs and bioactive scaffolds is the most effective therapy for heart failure patients. The proposed meta-analysis will aid in corroborating these findings, providing the necessary evidence for the use of bioactive scaffolds in the clinic.

There is a need for translational research that aids in bringing more novel and innovative therapies to the clinic. In this protocol, we have demonstrated the importance of using bioactive scaffolds for cardiac repair and provide a strategy for systematically reviewing the literature. The results of this meta-analysis can be used to bring scaffold-based stem cell therapies to the clinic, in hopes of providing heart failure patients with better health outcomes.

## Additional file

Additional file 1: PRISMA-P Checklist. This protocol was written in accordance with the PRISMA-P guidelines [26] and the line numbers are references in PRISMA-P checklist explicitly. (PDF 207 kb)

## Abbreviations

CPC: Cardiac progenitor cell
LVEF: Left ventricular ejection fraction
MI: Myocardial infarction
PRISMA: Preferred Reporting Items for Systematic Reviews and Meta-Analyses
SYRCLE: Systematic Review Centre for Laboratory Animal Experimentation
